# Potassium wasting nephropathy in the setting of tizanidine overdose: a case report

**DOI:** 10.1186/s13256-021-02811-8

**Published:** 2021-05-01

**Authors:** Michael J. Brucculeri, Juan Garcia

**Affiliations:** Section of Nephrology, University of South Florida Morsani College of Medicine–Morton Plant Hospital, 300 Pinellas Street, Clearwater, FL 33756 USA

**Keywords:** Tizanidine, Potassium, Hypokalemia, Nephropathy, QT prolongation, Case report

## Abstract

**Background:**

Hypokalemia has been rarely attributed to tizanidine, although the precise mechanism is unclear. Severe hypokalemia is a well-established cause of abnormalities involving cardiac conduction. Given this agent’s well-known cardiac arrhythmogenic potential, awareness of potential concomitant electrolyte abnormalities is important.

**Case presentation:**

Electrolyte disorders, including hypokalemia, are rare complications of the antispasmodic medicine tizanidine when taken in doses as outlined by the manufacturer’s prescribing instructions. Although cases of severe hypokalemia have also been described in the literature in association with this agent, such reports are few. We report a Caucasian case of an intentional overdose involving a very large ingestion of tizanidine. In addition to the characteristic abnormalities on the electrocardiogram, our patient developed electrolyte derangements as well as self-limited acute kidney injury. These biochemical abnormalities included profound hypokalemia that was refractory to aggressive replacement over the ensuing several days, before eventually dissipating. A thorough assessment of the etiology of this hypokalemia was consistent with a defect in renal potassium handling.

**Conclusion:**

In our patient with intentional tizanidine overdose, severe and refractory hypokalemia appears to have been due to a transient potassium wasting nephropathy.

## Introduction

Drug-induced hypokalemia is a complication often encountered in clinical practice, caused by a variety of therapeutics. Typically, these cases are mild and the patients respond easily to oral and/or intravenous potassium supplementation. Potassium wasting nephropathies, however, are far more unusual and difficult to treat as ongoing renal potassium losses can outstrip replacement. These serious and often severe electrolyte disorders can lead to significant morbidity if not properly recognized and aggressively treated. Nowhere is this more apparent than the effect of severe hypokalemia on myocyte (and specifically myocardial) conduction abnormalities. Tizanidine, a frequently prescribed muscular relaxant and antispasmodic, has been described to result in hypokalemia. We report here a case of severe hypokalemia presenting as a potassium wasting nephropathy.

## Case presentation

**Fig. 1:**
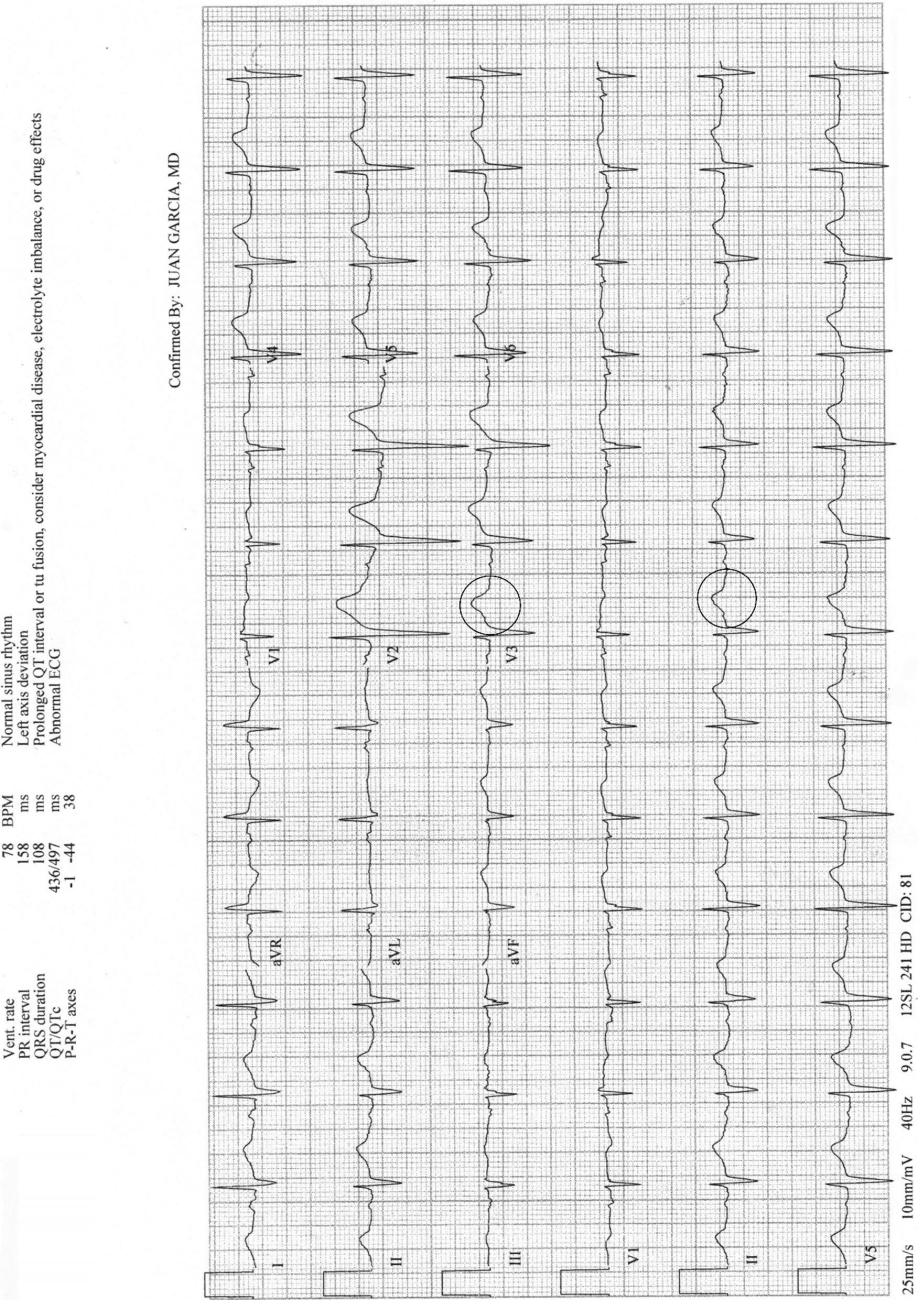
Initial electrocardiogram. Prolonged QTc interval of 497ms with T-U fusion waves (circled) most apparent in leads V3 and II

A 36-year-old Caucasian man with a past medical history significant for hypertension, depression, substance abuse, and dyslipidemia presented to the hospital after an intentional overdose with tizanidine. He was found by a friend in a state of reduced consciousness and severe weakness. He had consumed alcohol the night prior to presentation and had been vomiting profusely per report. In line with his presention, there were several abnormalities on his chemistry panel, including severe hypokalemia, hypomagnesemia, and acute kidney injury. Initial potassium concentration was 2.7 mmol/L; sodium concentration, 140 mmol/L magnesium, 1.4 mg/dL; and creatinine, 1.4 mg/dL (with a previously noted baseline of 0.8 mg/dL). Initial blood pressure was 105/68 mm/Hg, pulse was 82 bpm, and temperature was 98.5 degrees Fahrenheit. On physical exam he appeared to be comfortable and was cooperative. His head was atraumatic, and pupils were equal, round, and reactive to light. He had flat neck veins, and the lungs were clear to auscultation anteriorly and laterally. His abdomen was soft with normal active bowel sounds. He had no dependent edema. He had reduced muscle strength in both upper and lower extremities in a symmetrical fashion and had reduced patellar reflexes bilaterally. Results from an electrocardiogram were abnormal. He was found to have mild prolongation of the QT interval (corrected QT [QTc] 497 ms) with the presence of U waves having the appearance of T-U fusion waves (Fig. 1). Home medications included atorvastatin, levothyroxine, cetirizine, lisinopril, and tizanidine 4 mg every 6 h as needed.

He was admitted to the medical intensive care unit with continuous monitoring of his cardiac telemetry. The inpatient nephrology service was promptly consulted. He was provided with aggressive intravenous fluid resuscitation with isotonic crystalloid (lactated Ringers + 40 meq KCl/L at 125 ml/h); additional potassium replacement began in earnest both orally and parenterally. He was also administered magnesium sulfate 4 gm intravenously. Serial chemistries were ordered every 4 h. Further laboratory testing revealed an ethanol level of 219 mg/dL; acetaminophen, salicylates, and tricyclics were undectable. The urine drug screen was positive for cocaine. With further hydration and electrolyte replacement, his sensorium improved and he revealed that he had ingested 80 mg of tizanidine with 24 ounces of beer approximately 20 h before his initial presentation to the Emergency Department. The cocaine use was nearly a week prior to his presentation.

**Fig. 2:**
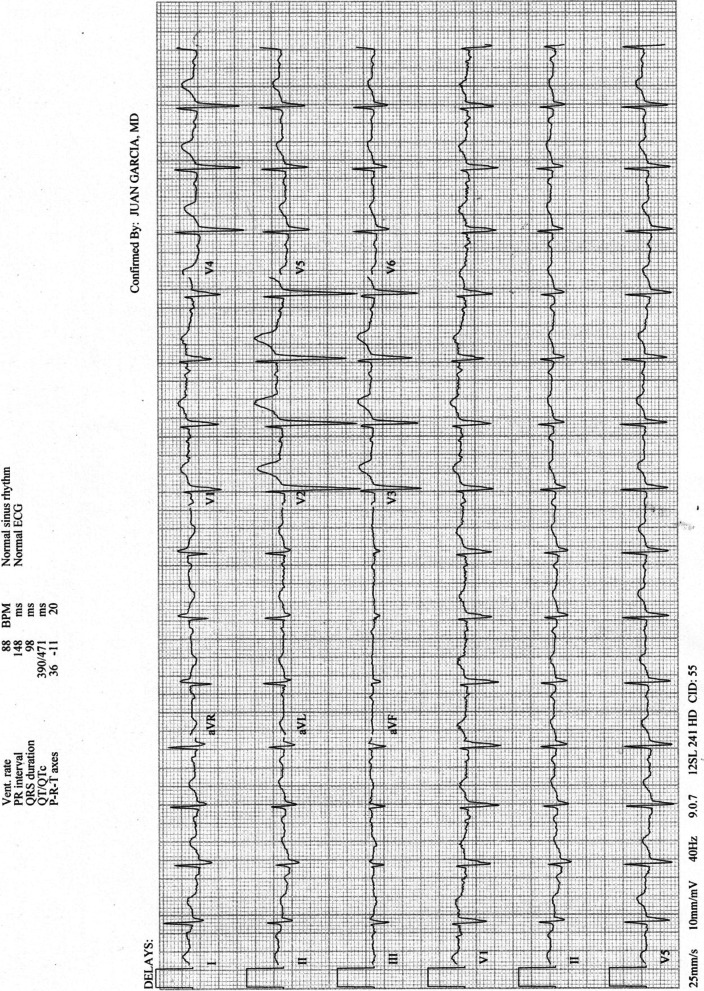
Final electrocardiogram on hospital day 4 after potassium levels had stabilized. QTc was now 471 with resolution of previously noted T and U wave abnormalities

Twenty-four hours after admission, after having received 100 mEq of potassium chloride his potassium remained extremely low at 2.8 mmol/L. At this point, the serum magnesium level had improved to 1.9 mg/dL. Repeat ECG confirmed ongoing mild QT prolongation with QTc of 497 ms. The patient’s urine potassium level was abnormally elevated at 38 mEq/L (should be < 20 mEq/L in the setting of severe hypokalemia), alluding to a defect in renal potassium handling, and not losses of gastrointestinal secretions which would have led to renal potassium conservation. Since he had achieved resolution of his acute kidney injury (creatinine was now down to 0.81 mg/dL), oral spironolactone 25 mg twice daily was started. The patient was transferred out of the intensive care unit to the cardiac step-down unit for ongoing monitoring of his telemetry and rhythm. Despite further improvement in his magnesium levels (> 2.0 mg/dL), his hypokalemia persisted for approximately 72 h more, during which time he required > 100 mEq daily, totaling nearly 500 meq. The potassium levels finally stabilized at 4.5 mmol/L, and the final ECG confirmed normalization of the QT interval with QTc of 471 ms (Fig. 2). On hospital day 5 he was transferred to the inpatient psychiatry service, at which time his electrolyte levels and renal function were normal. He was maintained on spironolactone 25 mg twice daily with no supplemental potassium requirements. Shortly after discharge, the patient’s aldosterone level returned to 3 ng/dL. The aldosterone to plasma renin activity ratio was reported as 7.7, which is not suggestive of primary hyperaldosteronism.

## Discussion

Tizanidine is a commonly prescribed agent for muscle spasm. It is a centrally acting alpha-2 adrenergic agonist leading to increased pre-synaptic motor neuron inhibition, thereby reducing spasticity [1]. Use of tizanidine has been well reported to cause bradycardia, QT prolongation, and Torsades de Pointes [2, 3]. As detailed in the case presentation, our patient did show a modest increase of the QT interval.

Hypokalemia attributed to tizanidine use has also been described and is listed on the prescribing information as a rare adverse event when taken as directed and not in total daily doses exceeding 24 mg [4], although information on severe cases in the literature is sparse [5, 6]. Once ingested, 60% of tizanidine is eliminated by the kidneys, and in those with a renal impairment (such as our patient), it is expected that exposure to the drug increases. It has been suggested that the mechanism by which tizanidine results in hypokalemia is through a defect in renal potassium handling, which was confirmed in our case. In the setting of profound hypokalemia, a urine random potassium level of < 20 mmol/L is generally considered an appropriate renal tubular response [7]. Other authors have purported that the use of the urine random potassium to creatinine ratio as a superior measure as this method corrects for day to day vacillations in urinary volume [7, 8]. These authors report that a urine random potassium level of > 20 mEq/g of creatinine is suggestive of renal potassium wasting; by this measure our patient was excreting 78 mEq/g of creatinine following his ingestion of tizanidine. Finally, not surprisingly, our patient had a trans-tubular potassium gradient (TTKG) value of 5; in the setting of clinically significant hypokalemia it should be < 2 [9]. It is conceivable that the ethanol consumption/abuse may have been a contributing factor to the development of marked hypokalemia. Hypokalemia in alcoholics is a well-described phenomenon that is attributed to gastrointestinal losses as well as kaliuresis in the setting of hypomagnesemia which is often chronic in nature and difficult to treat. The mechanism of hypokalemia in magnesium deficiency, however, remains an area of ongoing investigation. Magnesium deficiency has been shown to affect the function of the distal tubular epithelial cells in the nephron. Hypomagnesemia appears to modulate both sodium–potassium adenosine triphosphatase pump (Na-K-ATPase) function and the basolateral membrane as well as the flow of potassium out of the apical renal outer medullary potassium channel (ROMK) channels leading to enhanced renal K^+^ excretion [10]. Accordingly, parenteral magnesium administration appears to decrease urinary K^+^ excretion in individuals with hypokalemia as well as in normal subjects [10, 11].

Our patient did not have a history of chronic ethanol abuse, although he did have concomitant hypomagnesemia on presentation. It is unlikely that he had a chronic magnesium deficiency as his hypomagnesemia was easily replaced and he was able to maintain normal serum magnesium levels. Despite this, the potassium wasting persisted as detailed above. It should be noted that the Naranjo probability scale for adverse drug reactions indicates that tizanidine was the probable cause of the potassium wasting nephropathy in this patient [12].

## Conclusion

The implications in our findings suggest that an important mechanism by which tizanidine may result in severe hypokalemia is through a transient renal potassium wasting syndrome. Accordingly, it is of paramount importance to screen for hypokalemia and treat this and other electrolyte derangements (namely, hypomagnesemia which often coexists) aggressively in this clinical context. It is interesting that although our patient had significant hypomagnesemia upon presentation, this was remedied, and magnesium levels quickly normalized within hours. Hypokalemia (due to ongoing renal potassium wasting) and subsequent QT prolongation took a much longer duration (several days) to improve.

In summary, we describe a man with intentional tizanidine overdose who developed acute kidney injury, severe electrolyte disturbances, including marked hypokalemia, as well as QT prolongation (on ECG). Our patient confirmed the presence of a transient defect in renal potassium handling that led to renal potassium wasting. Fortunately, with supportive measures, his renal indices improved, and potassium wasting nephropathy was self-limited.

## Data Availability

Not applicable.

## Abbreviations

ECG: Electrocardiogram
Na^+^/K^+^-ATPase: Sodium–potassium adenosine triphosphatase pump
QT: QT interval
QTc: Corrected QT interval (for heart rate)
ROMK: Renal outer medullary potassium channel
TTKG: Trans-tubular potassium gradient
